# Age at Diagnosis and C-Peptide Level Are Associated with Diabetic Retinopathy in Chinese

**DOI:** 10.1371/journal.pone.0091174

**Published:** 2014-03-10

**Authors:** Xiaoling Cai, Xueyao Han, Simin Zhang, Yingying Luo, Yingli Chen, Linong Ji

**Affiliations:** Endocrinology & Metabolism Department, Peking University People's Hospital, Beijing, China; University of Florida, United States of America

## Abstract

**Objective:**

To find the associations between diabetic retinopathy and age at diagnosis, C-peptide level and thyroid-stimulating hormone (TSH) level in Chinese type 2 diabetes mellitus.

**Methods:**

3100 hospitalized type 2 diabetic patients in Peking University People's Hospital were included in this retrospective study. Their medical history and the laboratory data were collected. All the patients received examination of diabetic retinopathy (DR) by professional ophthalmologist.

**Results:**

Comparisons among patients with NDR, NPDR and PDR showed that with the progression of diabetic retinopathy, patients turned to have older age but younger age at diagnosis of diabetes, and have higher SBP, longer duration of diabetes, higher mean HbA1c but lower fasting and 2 hours postprandial C-peptide level. Moreover, with the progression of diabetic retinopathy, patients turned to have higher prevalence of primary hypertension, higher prevalence of peripheral vascular sclerosis, higher proportion with insulin treatment. TSH level was comparable among the three groups of patients. Association analysis showed that after adjusting for age, sex, duration of diabetes, body mass index, HbA1c, blood pressure and albuminurea creatinine ratio and insulin treatment, age at diagnosis (OR 0.888, 95%CI 0.870–0.907, p = 0.00) and postprandial C-peptide (OR 0.920, 95%CI 0.859–0.937, p = 0.00) are the independent associated factors of DR in Chinese type 2 diabetes.

**Conclusions:**

According to the results, postprandial C-peptide level and age at diabetes may be two independent associated factors with DR in Chinese type 2 diabetes. The lower level of postprandial C-peptide, the younger age at diagnosis, may indicate the higher prevalence of DR.

## Introduction

The prevalence of type 2 diabetes mellitus has increased worldwide [1], especially in the Asia-Pacific region [2] and China [3]. Consequently, diabetic retinopathy, one of the microvascular complications of diabetes, remains a major cause of visual impairment and blindness in the Asia- Pacific region [4]. The prevalence of diabetic retinopathy in China has been reported in several studies recently and the associated factors were also analysed in these studies, varying in size, design, and complexity. As concluded by several studies, age, duration of diabetes, level of glycemic control, blood pressure level, microalbuminuria, were demonstrated as the associated factors with diabetic retinopathy [2], [5]–[7]. With the realization of diabetic retinopathy, several new factors have been convinced by researchers associated with diabetic retinopathy in type 2 diabetes patients.

Thomas RL [8] reported in 2012 that age at diagnosis of diabetes was independently associated with DR and PDR in United Kingdom. However, there is few studies focus on it. Mosier MA [9] and Subrata [10] indicated that in type 1 diabetes patients, patients with PDR and NPDR showed different level of C-peptide. The preservation of C-peptide level might be useful in preventing the incidence of diabetic retinopathy. The opposite voice came from Klein R in the Wisconsin Epidemiologic Study of Diabetic Retinopathy [11], which showed that there was no relationship between C-peptide level and DR. Other risk factors such as thyroid level that have been reported to be correlated with diabetic retinopathy also need to be demonstrated. Associations of the above new factors with DR were inconsistent, therefore, in the current study, we make three hypotheses, first one is that age at diagnosis of diabetes is inversely associated with DR in established Chinese type 2 diabetes population, the second one is that level of C-peptide is inversely associated with DR, and the third one is that there is an association between thyroid level and DR in Chinese diabetes patients.

The aim of this retrospective study was to evaluate the relationship between these factors and diabetic retinopathy.

## Materials and Methods

### Patients

By searching inpatient database at Peking University People's Hospital, we have identified 3100 type 2 diabetic patients who were hospitalized for treatment at the ward of Department of Endocrinology and Metabolism of Peking University People's Hospital from Jan. 2004 to Dec. 2011. Reasons for hospitalization were as followings: 1) Advancement of oral hypoglycemic agents treatment or insulin treatment for better glycemic control. 2) Optimizing the anti-diabetic treatment and the treatment of diabetes complications. 3) Routine screening for diabetes complications by the patients' will. Patients with diabetic ketoacidosis or ketonuria and tested positive for anti-glutamic acid decarboxylase and islet cell antibody were excluded.

### Ethics statement

The data were analysed anonymously in this retrospective study, therefore, there is no need for informed consent. The ethics committee of Peking University People's Hospital has approved this retrospective study. All the patients were patients admitted in the department of Endocrine & Metabolism of our hospital, and during the day of their admitted, they signed the consent form for allowing their information to be stored in the hospital database and used for research, and this consent form was also approved by the ethics committee of Peking University People's Hospital.

### Variable assessment

Anthropometric measurements at admission were collected, biochemical measurements including HbA1c (Primus ultra2, Primus Diagnostics, MO, USA), urinary albumin-creatinine ratio(COBAS Integra 400 Plus System, Roche Diagnostics Ltd. Basel, Switzerland), fasting and 2 hour postprandial insulin (Elecsys 2010 system, Roche Diagnostics Ltd, Basel, Switzerland), fasting and 2 hour postprandial C-peptide (ACS180,BYAER, Fribourg, Switzerland), levels of total cholesterol (CHO), low-density lipoprotein cholesterol (LDL-C), high-density lipoprotein cholesterol (HDL-C), and triglyceride (COBAS Integra 400 Plus System, Roche Diagnostics Ltd. Basel, Switzerland) were assessed. Uric Acid and serum creatinine were also assessed. Urinary albumin-to-creatinine ratio (ACR) was tested in three consecutive days for each patient, and the average ACR was calculated. Levels of thyroid function including free thyroxine (FT4), free triiodothyronine (FT3), thyroxine (TT4), triiodothyronine (TT3), Thyroid-stimulating hormone (TSH) were also tested (SIMENZ centaur xp). The peripheral vascular sclerosis was diagnosed by ultrasound of carotid artery and lower extremity artery.

### Diabetic retinopathy

The patients received retinal photography by the same trained photographer taking two 45° digital retinal images per eye (one macular centred, and one nasal field) using a TOPCON DGi camera. The retinal images then were transferred to the ophthalmologist for grading. Some confounding cases were finally diagnosed through fundus fluorescein angiography (FFA). Diabetic retinopathy was clinically graded according to the new diabetic retinopathy disease severity scale [12]. The results were defined as no apparent retinopathy (NDR), non-proliferative diabetic retinopathy (NPDR), and proliferative diabetic retinopathy (PDR). The ophthalmologist described each category of the funduscopic findings as follows: 1) No apparent retinopathy, no abnormalities; 2) mild NPDR, microaneury only; 3) moderate NPDR, more than just microaneurysms but less than severe nonproliferative diabetic retinopathy; 4) severe NPDR, any of the following: more than 20 intraretinal hemorrhages in each of 4 quadrants; definite venous beading in 2 quadrants; prominent intraretinal microvascular abnormalities in 1 quadrant and no signs of proliferative reinopathy; and 5) PDR, one or more of the following: neovascularization, vitreous/pre-retinal hemorrhage.

### Statistical analysis

Variables were represented as means ± s.d. Continuous variables were compared by using a one way analysis of variance (ANOVA) test while frequency of dichotomous variables was performed by χ^2^ analysis. A two-sided p≤0.05 was considered significant. Multivariable logistic regression analyses were made to assess the correlation between diabetic retinopathy and age at diagnosis of diabetes, level of C-peptide, or thyroid level. All P-values were 2-tailed and considered significant at P<0.05. Regression analyses were performed using SPSS 19.0.

## Results

### Prevalence of diabetic retinopathy in hospitalized patients and demographic data

The overall prevalence of diabetic retinopathy was 23.7% in these hospitalized patients with type 2 diabetes. The prevalence of NPDR was 18.2% and the prevalence of PDR was 5.5%. The prevalence of mild, moderate, and severe NPDR was 11.1%, 2.7%, and 4.4%, respectively. Of the patients in this study, 59.4% were males. The average age was 57.1±13.7 years, duration of diabetes was 9.14±7.80 years, average BMI was 25.0±3.7 kg/m^2^, average HbA1c was 9.26±2.23%. The age at diagnosis of diabetes was 48.0±12.3 years. The prevalence of primary hypertension was 51.1%, the prevalence of peripheral vascular sclerosis was 80.1%. In terms of antidiabetic treatment, the proportion of patients received oral hypoglycemic agent (OHA) was 62.6%, of patients received insulin treatment was 37.4%. The proportion of patients receiving anti-platelet treatment was 17.0%.

### Demographic data and metabolic profile of patients with DR

Comparisons among patients with NDR, NPDR and PDR showed that with the progression of diabetic retinopathy, patients turned to have older age but younger age at diagnosis of diabetes, and have higher SBP, longer duration of diabetes, higher mean HbA1c but lower fasting and 2 hours postprandial C-peptide level. Moreover, with the progression of diabetic retinopathy, patients turned to have higher prevalence of primary hypertension, higher prevalence of peripheral vascular sclerosis, higher proportion with insulin treatment. TSH level was comparable among the three groups of patients. Details were shown in Table 1.

**Table 1:** Comparisons between patients with NDR and DR, patients with NPDR and PDR.

	NDR	NPDR	PDR
N	1546	368	111
Male*	63.7%	49.3%	45.9%
Age (years)*	55.6±14.0	61.9±11.6	60.1±11.8
Age at diagnosis of diabetes (years)*	48.6±12.6	48.4±11.3	44.6±10.5
Duration of diabetes (years)*	7.4±6.8	13.5±7.7	15.5±7.9
BMI (kg/m^2^)	25.2±3.7	24.9±3.5	25.2±3.5
SBP (mmHg)*	130.3±17.7	140.0±18.9	145.1±19.6
DBP (mmHg)	79.3±10.7	79.6±10.9	81.9±12.5
HbA1c (%)*	9.2±2.2	9.5±2.1	9.0±2.1
Fasting C-peptide (ng/dl)	1.9±1.1	1.7±1.0	1.7±1.1
Postprandial C-peptide (ng/dl)*	4.4±2.9	3.4±2.2	3.2±2.1
UA(umol/L)*	305.9±88.8	315.0±95.8	342.5±90.0
CRE(umol/L)*	69.1±22.5	75.8±33.7	98.2±64.5
CHO (mmol/L)*	4.7±1.1	5.0±1.2	5.0±1.3
TG(mmol/L)	1.9±1.7	1.8±1.3	2.0±1.1
HDL-C (mmol/L)	1.1±0.3	1.2±0.4	1.1±0.3
LDL-C (mmol/L)	2.8±0.9	2.9±1.0	2.8±0.8
FT4 (pmol/L)	15.1±3.2	15.3±1.9	13.8±0.9
FT3 (pmol/L)	4.3±1.1	4.3±0.5	3.8±0.2
TSH (uIU/ml)	2.2±5.6	2.1±2.1	1.7±1.3
ACR (mg/g)*	57.6±237.7	359.4±953.6	645.6±1079.5
prevalence of primary hypertention (%)*	47.0	64.6	76.7
prevalence of peripheral vascular sclerosis (%)*	76.0	94.2	92.0
patients with insulin treatment (%)*	26.5	33.9	64.9
patients with anti-platlet treatment (%)*	16.1	16.0	31.0

*comparison among groups showed significant difference.

Abbreviations in the tables: NDR: non diabetic retinopathy; NPDR: non-proliferative diabetic retinopathy; PDR: proliferative diabetic retinopathy; BMI: body mass index; SBP: systolic blood pressure; DBP: diastolic blood pressure; HbA1c: hemoglobin A1c; UA: uric acid; CRE: creatinine; CHO: cholesterol; TG: triglyceride; HDL-C: high density lipoprotein cholesterol; LDL-C: low density lipoprotein cholesterol; FT4: free thyroxine; FT3: free triiodothyronine; TSH: Thyroid-stimulating hormone; ACR: urine albumin creatinine ratio.

### Association between age at diagnosis of diabetes and DR

Demographic data and metabolic profile of patients from lowest quartile to highest quartile of age at diagnosis of diabetes were shown in Table 2, which indicated that, with the decrease of age at diagnosis, patients turned to have longer duration of diabetes, lower level of fasting C-peptide and lower level of postprandial C-peptide, higher BMI, and younger age. With the decrease of age at diagnosis, patients turned to have higher prevalence of proliferative diabetic retinopathy. Association analysis between age at diagnosis and DR showed that after adjusted for confounding variables as gender, age, BMI, HbA1c, diabetic duration, SBP, ACR, serum creatinine and insulin treatment, age at diagnosis was negatively associated with DR (shown in Table 3). Association analysis between age at diagnosis and PDR showed that after adjusted for confounding variables, age at diagnosis was also negatively associated with PDR (OR 0.961, 95%CI 0.938–0.985, p<0.05).

**Table 2:** Comparisons between patients in different level of age at diagnosis.

	Lowest quartile	Second quartile	Third quartile	Highest quartile ^#^
age (years)*	43.9±11.9	53.4±8.1	60.9±7.8	71.3±6.7
Age at diagnosis of diabetes (years)*	33.7±6.1	44.1±2.1	51.4±2.2	64.1±6.4
Duration of diabetes (years)*	10.2±8.7	9.4±7.9	9.5±7.5	7.2±6.2
BMI (kg/m^2^)*	25.3±3.9	25.3±3.5	25.0±3.5	24.8±3.5
HbA1c (%)	9.4±2.2	9.2±2.1	9.0±2.2	9.4±2.4
Fasting C-peptide (ng/dl)*	1.64±1.0	1.71±0.85	1.85±0.98	1.98±1.14
Postprandial C-peptide (ng/dl)*	3.5±2.4	3.9±2.3	4.5±2.9	4.5±2.8
prevalence of primary hypertention (%)*	35	49	58	67
prevalence of DR (%)	25	26	21	22
prevalence of PDR (%)*	33	26	17	14
prevalence of peripheral vascular sclerosis (%)*	62	80	88	94
patients with insulin treatment (%)*	42	36	33	34

*comparison among groups showed significant difference.

#lowest quartile: age at diagnosis≤40 years old; second quartile: 40 years old <age at diagnosis≤48 years old; third quartile: 48 years old <age at diagnosis≤56 years old; highest quartile: 56 years old <age at diagnosis.

Abbreviations in the tables: DR: diabetic retinopathy; PDR: proliferative diabetic retinopathy; BMI: body mass index; HbA1c: hemoglobin A1c.

**Table 3:** Association of age at diagnosis, fasting C-peptide or postprandial C-peptide with diabetic retinopathy in type 2 diabetic patients by multivariate logistic regression analysis*.

	Model 1		Model 2		Model 3	
	Odds ratio	95% CI	Odds ratio	95% CI	Odds ratio	95% CI
age at diagnosis	0.895	0.880–0.910	0.886	0.870–0.902	0.888	0.870–0.907
postprandial C-peptide	0.847	0.805–0.892	0.889	0.837–0.944	0.920	0.859–0.937
fasting C-peptide	0.867	0.776–0.968	1.040	0.912–1.181	1.019	0.882–1.191

*Model 1: adjusted for gender and age. Model 2: adjusted for gender, age, BMI and HbA1c. Model 3: adjusted for gender, age, BMI, HbA1c, diabetic duration, SBP, ACR, creatinine and insulin treatment.

### Association between level of C-peptide and DR

Demographic data and metabolic profile of patients from lowest quartile to highest quartile of fasting C-peptide and postprandial C-peptide were shown in Table 4 and 5, which indicated that, with the decrease of fasting C-peptide and postprandial C-peptide, patients turned to have younger age at diagnosis of diabetes, longer duration of diabetes, lower BMI and higher HbA1c. With the decrease of fasting C-peptide and postprandial C-peptide, patients turned to have higher prevalence of diabetic retinopathy, but the prevalence of proliferative diabetic retinopathy was comparable among groups (shown in Table 4 and 5). Association analysis between fasting C-peptide or postprandial C-peptide and DR showed that after adjusted for confounding variables as gender, age, BMI, HbA1c, diabetic duration, SBP, ACR, serum creatinine and insulin treatment, postprandial C-peptide was negatively associated with DR (shown in Table 3).

**Table 4:** Comparisons between patients in different level of fasting C-peptide.

	Lowest quartile	Second quartile	Third quartile	Highest quartile^#^
age (years)	57.0±14.8	56.0±13.1	57.4±12.3	57.4±13.8
Age at diagnosis of diabetes (years)*	46.5±12.8	46.8±11.4	48.9±11.4	50.0±12.3
Duration of diabetes (years)*	10.4±8.4	9.2±7.9	8.5±6.9	7.4±6.7
BMI (kg/m^2^)*	23.1±3.0	24.9±3.3	25.7±3.1	26.7±3.9
HbA1c (%)*	10.0±2.4	9.5±2.1	9.1±2.1	8.7±2.0
Fasting C-peptide (ng/dl)*	0.76±0.21	1.32±0.14	1.86±0.18	3.06±0.97
Postprandial C-peptide (ng/dl)*	1.93±1.23	3.13±1.43	4.30±1.98	6.60±2.83
prevalence of primary hypertention (%)*	40	49	52	62
prevalence of DR (%)*	29	20	20	24
prevalence of PDR (%)	23	25	20	24
prevalence of peripheral vascular sclerosis (%)	83	78	81	79
patients with insulin treatment (%)*	44	37	35	29

*comparison among groups showed significant difference.

#lowest quartile: fasting C-peptide≤1.06 ng/dl; second quartile: 1.06 ng/dl < fasting C-peptide≤1.55 ng/dl; third quartile: 1.55 ng/dl < fasting C-peptide≤2.19 ng/dl; highest quartile: 2.19 ng/dl < fasting C-peptide.

Abbreviations in the tables: DR: diabetic retinopathy; PDR: proliferative diabetic retinopathy; BMI: body mass index; HbA1c: hemoglobin A1c.

**Table 5:** Comparisons between patients in different level of postprandial C-peptide.

	Lowest quartile	Second quartile	Third quartile	Highest quartile^#^
age (years)	57.6±14.6	57.3±13.3	57.1±13.0	58.0±12.4
Age at diagnosis of diabetes (years)*	46.5±13.1	47.4±11.7	48.6±11.6	50.5±11.6
Duration of diabetes (years)*	11.0±8.9	9.9±7.6	8.5±7.4	7.6±6.1
BMI (kg/m^2^)*	23.4±3.1	25.1±3.1	26.0±3.4	26.3±3.8
HbA1c (%)*	10.3±2.4	9.5±2.0	9.1±2.0	8.2±1.8
Fasting C-peptide (ng/dl)*	0.97±0.42	1.52±0.55	1.91±0.69	2.78±1.18
Postprandial C-peptide (ng/dl)*	1.40±0.45	2.71±0.38	4.23±0.55	7.75±2.14
prevalence of primary hypertention (%)*	45	50	54	63
prevalence of DR (%)*	34	26	21	15
prevalence of PDR (%)	23	29	19	23
prevalence of peripheral vascular sclerosis (%)	80	82	81	80
patients with insulin treatment (%)*	55	54	41	29

*comparison among groups showed significant difference.

#lowest quartile: postprandial C-peptide≤2.1 ng/dl; second quartile: 2.1 ng/dl < postprandial C-peptide≤3.41 ng/dl; third quartile: 3.41 ng/dl < postprandial C-peptide≤5.33 ng/dl; highest quartile: 5.33 ng/dl < postprandial C-peptide.

Abbreviations in the tables: DR: diabetic retinopathy; PDR: proliferative diabetic retinopathy; BMI: body mass index; HbA1c: hemoglobin A1c.

### Association between level of TSH and DR

Comparisons between patients with higher and lower level of TSH indicated that patients with lower level of TSH showed lower level of ACR (59.8±324.7 mg/g vs 220.7±721.5, P<0.01), while other variables were all comparable between groups. Association analysis showed that TSH level was not associated with DR.

## Discussion

### Prevalence of diabetic retinopathy

Previous individual studies have shown considerable variability in DR prevalence estimates among individuals with diagnosed diabetes, with rates ranging from 17.6% in a study in India [13] to 33.2% in a large U.S. study [14]. In a meta-analysis included a total of 35 studies provided data from 22,896 individuals with diabetes, it indicated that the overall prevalence was 34.6% (95% CI 34.5–34.8) for any DR, 6.96% (6.87–7.04) for proliferative DR [15]. A recent published study in UK [16] indicated that in type 2 diabetes, the prevalence of any DR was 38.0% in white Europeans compared to 52.4% in African/Afro-Caribbeans and 42.3% in South Asians, which indicated more higher prevalence of DR. In Asian patients, two recently published papers have been found. One is in Korean patients [7], which showed the overall prevalence of diabetic retinopathy in the population was 18% and proliferative or severe non-proliferative form was found in 5.0% of the study subjects, the other is a meta-analysis in mainland China [17], which showed that the prevalence of DR, NPDR and PDR in the diabetic group was 23%, 19.1%, and 2.8%. According to our hospital-based data from this study, the prevalence of diabetic retinopathy in Chinese was 23.57%, and 5.5% for PDR.

### Age at diagnosis and diabetic retinopathy

A few studies had indicated that younger onset of diabetes was associated with DR. In an Asian survey data, they found that photocoagulation, retinopathy, and advanced eye disease were more common in patients diagnosed before the age of 30 than those after the age of 30 [18]. A study of young Japanese type 2 diabetes patients also revealed 13% were diagnosed with proliferative retinopathy before the age of 35 [19]. Thomas RL [8] reported that in United Kingdom the incidence of referable retinopathy was inversely related to age at diagnosis. In another UK study, younger onset diabetes patients had higher rates of retinopathy after accounting for diabetes duration [20]. According to our hospital-based data from this study, age at diagnosis of diabetes was negatively associated with DR in Chinese patients. It was suggested that patients with young onset of diabetes had longer disease duration and therefore higher rates of retinopathy. Another reason might be young onset of diabetes patients were less likely to meet guideline-recommended since they may consider controlling risk factors at their age as a legacy thing.

### C-peptide level and diabetic retinopathy

C-peptide was considered to be a biologically inert portion of proinsulin. Fasting C-peptide level was found to be associated with a reduced incidence of microvascular complications in type 1 diabetes [21]. In contrast, the role of C-peptide is not well defined in type 2 diabetes, of which insulin resistance and insulin secretion defect both exist. Some studies found that C-peptide level was a protective effect on microvascular complications. A study from Korean [22] indicated that in the patients with lower delta C-peptide (postprandial – fasting C-peptide) quartile, the prevalence of diabetic retinopathy was significantly higher (P<0.001) and low delta C-peptide quartile was also associated with increased severity of retinopathy. Other studies in Asian also demonstrated the association between C-peptide level and diabetic retinopathy[23]–[25].A recent study in European [26] also indicated that the risks for incident retinopathy was negatively associated with the highest C-peptide tertile, after adjusting for multiple confounders (hazard ratio (HR) = 0.33; 95% CI 0.23–0.47). While others failed to find such an association [27], [28]. In Australian Population, factors independently associated with retinopathy were duration of diabetes, HbA_1c_, and systolic blood pressure but not C-peptide [29]. What we have found in our study was that postprandial C-peptide level was associated with diabetic retinopathy, which was in concordance with that found in Asians.

### TSH level and diabetic retinopathy

Yang indicated that the trend for severe retinopathy was significantly higher in the subclinical hypothyroidism (SCH) group than in the euthyroid group. SCH was associated with greater prevalence of diabetic retinopathy [30]. Kim [31] reported that SCH was independently associated with severe diabetic retinopathy in patients with type 2 diabetes and the prevalence of severe diabetic retinopathy was significantly higher in the subclinical hypothyroidism group than the euthyroid group (32.8% *vs*. 19.6%, *P* = 0.036). On the contrary, Ramis reported that they could not find any relationship between either TSH levels or the presence of SCH and DR in Caucasian population [32]. Chen [33] reported that patients with type 2 diabetes and SCH were at increased risk of nephropathy and cardiovascular events, but not retinopathy. Our results were in consistent with the negative association one. Reasons for this discrepancy are unclear at present, but they may be related to differences in study design, characteristics of the participants, and ethnicity. Our study included a larger hospital-based population than the other studies and all the patients underwent a professional fundus examination.

### Limitations

Our study had some limitations. First, this study was designed as a retrospective study, in which the causation of diabetic retinopathy could not be directly assessed. Therefore, results concluded from this study just indicated some predictors for DR. Second, patients included in this study were hospital-based, in which there might be a selection bias. Furthermore, some confounding factors may also exist such as osteoporosis. Whatever, this is a study focused on some factors that may be associated with diabetic retinopathy but seldom being studied previously. What's more, the number of patients included in this study was a larger one than previously reported, which may indicate a more convincing result.

## Conclusion

According to the results of these hospitalization-based population, age at diagnosis of diabetes and postprandial C-peptide level may be two independent associated factors with DR in Chinese type 2 diabetes. The younger age at diagnosis, the lower level of postprandial C-peptide may indicate the higher prevalence of DR.

## References

[1] King H, Aubert RE, Herman WH (1998) Global burden of diabetes, 1995–2025: prevalence, numerical estimates, and projections. Diabetes Care 21: 1414–31. 9727886 10.2337/diacare.21.9.1414

[2] Chan JC, Malik V, Jia W, Kadowaki T, Yajnik CS, et al (2009) Diabetes in Asia: epidemiology, risk factors, and pathophysiology. JAMA 301: 2129–40. 19470990 10.1001/jama.2009.726

[3] Yang W, Lu J, Weng J, Jia W, Ji L, et al (2010) China National Diabetes and Metabolic Disorders Study Group. Prevalence of diabetes among men and women in China. N Engl J Med 362(12): 1090–101. 20335585 10.1056/NEJMoa0908292

[4] Porta M, Bandello F (2002) Diabetic retinopathy: a clinical update. Diabetologia 45: 1617–34. 12488951 10.1007/s00125-002-0990-7

[5] Kostev K, Rathmann W (2013) Diabetic retinopathy at diagnosis of type 2 diabetes in the UK: a database analysis. Diabetologia 56(1): 109–11. 23052061 10.1007/s00125-012-2742-7

[6] Cai XL, Wang F, Ji LN (2006) Risk factors of diabetic retinopathy in type 2 diabetic patients. . Chin. Med. J. 119(10): 822–826. 16732984

[7] Kim JH, Kwon HS, Park YM, Lee JH, Kim MS, et al (2011) Prevalence and Associated Factors of Diabetic Retinopathy in Rural Korea: The Chungju Metabolic Disease Cohort Study. J Korean Med Sci 26: 1068–1073. 21860558 10.3346/jkms.2011.26.8.1068 PMC3154343

[8] Thomas RL, Dunstan F, Luzio SD, Roy Chowdury S, Hale SL, et al (2012) Incidence of diabetic retinopathy in people with type 2 diabetes mellitus attending the Diabetic Retinopathy Screening Service for Wales: retrospective analysis. BMJ 344: e874. 22362115 10.1136/bmj.e874 PMC3284424

[9] Mosier MA (1984) Circulating C-peptide and diabetic retinopathy. Diabetes Res 1(3): 151–4. 6529887

[10] Nakanishi K, Watanabe C (2008) Rate of beta-cell destruction in type 1 diabetes influences the development of diabetic retinopathy: protective effect of residual beta-cell function for more than 10 years. J Clin Endocrinol Metab 93(12): 4759–66. 18826998 10.1210/jc.2008-1209

[11] Klein R, Klein BE, Moss SE (1995) The Wisconsin Epidemiologic Study of Diabetic Retinopathy. XVI. The relationship of C-peptide to the incidence and progression of diabetic retinopathy. Diabetes 44(7): 796–801. 7789648 10.2337/diab.44.7.796

[12] Wilkinson CP, Ferris FL, Klein RE, Lee PP, Agardh CD, et al (2003) Global Diabetic Retinopathy Project Group. Proposed international clinical diabetic retinopathy and diabetic macular edema disease severity scales. Ophthalmology 110: 1677–82. 13129861 10.1016/S0161-6420(03)00475-5

[13] Rema M, Premkumar S, Anitha B, Deepa R, Pradeepa R, et al (2005) Prevalence of diabetic retinopathy in urban India: the Chennai Urban Rural Epidemiology Study (CURES) eye study, I. Invest Ophthalmol Vis Sci 46: 2328–2333. 15980218 10.1167/iovs.05-0019

[14] Wong TY, Klein R, IslamFM, Cotch MF, Folsom AR, et al (2006) Diabetic retinopathy in a multi-ethnic cohort in the United States. Am J Ophthalmol 141: 446–455. 16490489 10.1016/j.ajo.2005.08.063 PMC2246042

[15] Yau JWY, Rogers SL, Kawasaki R, Lamoureux EL, Kowalski JW, et al (2012) Global Prevalence and Major Risk Factors of Diabetic Retinopathy. Diabetes Care 35: 556–564. 22301125 10.2337/dc11-1909 PMC3322721

[16] Sivaprasad S, Gupta B, Gulliford MC, Dodhia H, Mohamed M, et al (2012) Ethnic Variations in the Prevalence of Diabetic Retinopathy in People with Diabetes Attending Screening in the United Kingdom (DRIVE UK). PLoS ONE 7(3): e32182. 22412857 10.1371/journal.pone.0032182 PMC3297598

[17] Liu L, Wu X, Liu L, Geng J, Yuan Z, et al (2012) Prevalence of Diabetic Retinopathy in Mainland China: A Meta-Analysis. PLoS ONE 7(9): e45264. 23028893 10.1371/journal.pone.0045264 PMC3447927

[18] Chuang LM, Soegondo S, Soewondo P, Young-Seol K, Mohamed M, et al (2006) Comparisons of the outcomes on control, type of management and complications status in early onset and late onset type 2 diabetes in Asia. Diabetes Res Clin Pract 71(2): 146–55. 16005097 10.1016/j.diabres.2005.05.007

[19] Yokoyama H, Okudaira M, Otani T, Takaike H, Miura J, et al (1997) Existence of early-onset NIDDM Japanese demonstrating severe diabetic complications. Diabetes Care 20(5): 844–7. 9135953 10.2337/diacare.20.5.844

[20] Song SH, Gray TA (2011) Early-onset type 2 diabetes: high risk for premature diabetic retinopathy. Diabetes Res Clin Pract 94(2): 207–11. 21855159 10.1016/j.diabres.2011.07.030

[21] Steffes MW, Sibley S, Jackson M, Thomas W (2003) b-Cell function and the development of diabetes-related complications in the Diabetes Control and Complications Trial. Diabetes Care 26: 832–836. 10.2337/diacare.26.3.832 12610045

[22] Kim BY, Jung CH, Mok JO, Kang SK, Kim CH (2012) Association between serum C-peptide levels and chronic microvascular complications in Korean type 2 diabeticpatients. Acta Diabetol 49(1): 9–15. 21212993 10.1007/s00592-010-0249-6

[23] Zheng W (2011) Factor analysis of diabetic retinopathy in Chinese patients. Dia Res &Clin Prac 92: 244–252. 10.1016/j.diabres.2011.02.007 21388700

[24] Yoon HJ, Cho YZ, Kim J, Kim BJ, Park KY, et al (2012) Correlations between Glucagon Stimulated C-peptide Levels and Microvascular Complications in Type 2 Diabetes Patients. Diabetes Metab J 36: 379–387. 23130323 10.4093/dmj.2012.36.5.379 PMC3486985

[25] Fukui T, Oono K, Hara N, Yamamoto T, Nagashima M, et al. (2013) Increment of C-peptide after glucagon injection determines the progressive nature of Japanese type 2 diabetes: A long-term follow-up study. Endocrine Journal 60 (6): , 715–724. 10.1507/endocrj.ej12-0357 23386398

[26] Bo S, Gentile L, Castiglione A, Prandi V, Canil S, et al (2012) C-peptide and the risk for incident complications and mortality in type 2 diabetic patients: a retrospective cohort study after a 14-year follow-up. Eur J Endocrinol 167(2): 173–80. 22577110 10.1530/EJE-12-0085

[27] Klein R, Klein BE, Moss SE (1995) TheWisconsin Epidemiologic Study of diabetic retinopathy, XVI. The relationship of C-peptide to the incidence and progression of diabetic retinopathy. Diabetes 44: 796–801. 7789648 10.2337/diab.44.7.796

[28] Gottsater A, Ahmed M, Fernlund P, Sundkvist G (1999) Autonomic neuropathy in type-2 diabetic patients is associated with hyperinsulinemia and hypertriglyceridemia. Diabetic Medicine 16: 49–54. 10229293 10.1046/j.1464-5491.1999.00001.x

[29] Tapp RJ, Shaw JE, Harper CA, de Courten MP, Balkau B, et al (2003) The Prevalence of and Factors Associated With Diabetic Retinopathy in the Australian Population. Diabetes Care 26(6): 1731–7. 12766102 10.2337/diacare.26.6.1731

[30] Yang JK, Liu W, Shi J, Li YB (2010) An Association Between Subclinical Hypothyroidism and Sight-Threatening Diabetic Retinopathy in Type 2 Diabetic Patients. Diabetes Care 33: 1018–1020. 20150298 10.2337/dc09-1784 PMC2858165

[31] Kim BY, Kim CH, Jung CH, Mok JO, Suh KI, et al. (2011) Association between subclinical hypothyroidism and severe diabetic retinopathy in Korean patients with type 2 diabetes. Endocrine Journal 58 (12): , 1065–1070. 10.1507/endocrj.ej11-0199 21931224

[32] Ramis JN, Artigas CF, Santiago MA, Mañes FJ, Canonge RS, et al (2012) Is there a relationship between TSH levels and diabetic retinopathy in the Caucasian population? Diabetes Res Clin Pract 97(3): e45–7. 22717499 10.1016/j.diabres.2012.05.015

[33] Chen HS, Wu TE, Jap TS, Lu RA, Wang ML, et al (2007) Subclinical hypothyroidism is a risk factor for nephropathy and cardiovascular diseases in Type 2 diabetic patients. Diabet Med 24: 1336–1344. 17941864 10.1111/j.1464-5491.2007.02270.x

